# Perceived Academic Stress and Depression: The Mediation Role of Mobile Phone Addiction and Sleep Quality

**DOI:** 10.3389/fpubh.2022.760387

**Published:** 2022-01-25

**Authors:** Xin Zhang, Fei Gao, Zheng Kang, Hongguo Zhou, Jianfeng Zhang, Jingjing Li, Jun Yan, Jiahui Wang, Huan Liu, Qunhong Wu, Baohua Liu

**Affiliations:** ^1^Department of Social Medicine, School of Public Health, Health Management College, Harbin Medical University, Harbin, China; ^2^Institute of Food Safety and School Health, Heilongjiang Center for Disease Control and Prevention, Harbin, China; ^3^Department of Educational Administration, Ningbo College of Health Sciences, Ningbo, China; ^4^Department of Elderly Healthcare and Management, School of Health Services and Management, Ningbo College of Health Sciences, Ningbo, China

**Keywords:** perceived academic stress, mobile phone addiction (MPA), sleep quality, depression, depressive symptoms, Chinese students

## Abstract

**Background:**

Although academic stress is a well-known risk factor for students' depression, little is known about the possible psychological mechanisms underlying this association. In this study, we investigated the prevalence of depression and sleep disturbance among Chinese students, examined the relationship between perceived academic stress and depression, considered if mobile phone addiction and sleep quality is a mediator of this relationship, and tested if mobile phone addiction and sleep quality together play a serial mediating role in the influence of perceived academic stress on depression.

**Method:**

A cross-sectional survey was conducted among students from September to December 2018 in Heilongjiang Province, China. The final analysis included 5,109 students. Mobile phone addiction, sleep quality, and depressive symptoms were assessed using the Mobile Phone Addiction Index, Pittsburgh Sleep Quality Index, and Center for Epidemiologic Studies-Depression scales, respectively. The serial mediation model was used to analyse the relationship between perceived academic stress, mobile phone addiction, sleep quality, and depression.

**Results:**

Among all participants, the prevalence of depressive symptoms and sleep disturbance was 28.69 and 27.95%, respectively. High school students showed the highest scores of perceived academic stress (2.68 ± 1.06), and the highest prevalence of depressive symptoms (33.14%) and sleep disturbance (36.47%). The serial mediation model indicated that perceived academic stress was a significant predictor of depression (B = 0.10, SE = 0.02, 95% CI = 0.06 – 0.13). Additionally, mobile phone addiction (B = 0.08, 95% boot CI = 0.06–0.11) and sleep quality (B = 0.27, 95% boot CI = 0.22–0.33) played a mediating role between perceived academic stress and depression. Mobile phone addiction and sleep quality together played a serial mediating role in the influence of perceived academic stress on depression (B = 0.11, 95% boot CI = 0.08–0.14). Furthermore, the indirect effect (i.e., the mediating effect of mobile phone addiction and sleep quality) was significant and accounted for 64.01% of the total effect.

**Conclusions:**

Our research results underscore the need for stakeholders—including family members, educators, and policy makers—to take preventative intervention measures to address depression among Chinese students, especially high school students.

## Highlights

- Perceived academic stress significantly predicts depression.
- Sleep quality mediates perceived academic stress and depression.
- Mobile phone addiction mediates perceived academic stress and depression.
- Mobile phone addiction and sleep quality together play a serially mediating role in the influence of PAS on depression.

## Introduction

Depression (major depressive disorder) is a widespread chronic medical illness that can influence mood, thoughts, and physical health (1), and is a severe problem faced by students worldwide. A meta-analysis that included 183 studies from 43 countries shows that the overall pooled crude prevalence of depression was 27.2% among medical students (2). Previous studies demonstrated that the prevalence of depression was 51.3, 38.3, 28.4, and 30.6% among Indian students (3), Japanese adolescents (4), Chinese university students (5), and Cameroon medical students (6), respectively. It is important to evaluate the prevalence of depressive symptoms and explore the effect mechanism of depressive symptoms to protect students from the harmful effects of depression. Studies related to students' depressive symptoms often focus on a particular group of students, such as medical (2), college (7), and university students (8), and scant research exists about depressive symptoms among students at different levels of education. Many risk factors have been associated with depression, including being female (9, 10), life stressors (9, 10), physical and mental factors, social media addiction (11), and parental factors, including parental psychopathology and parenting attachment (12). Stress has been shown to be one of the most important risk factors of depression, and numerous studies have demonstrated that stress plays an important role in the emergence of depression (13–15). For example, Torres-Berrío et al. supposed that depression is caused by a combination of genetic predisposition and life events (16). Stress often leads to adverse consequences—such as depression and anxiety (17–19), mobile phone addiction (MPA) (20, 21), poor sleep quality (PSQ) (22, 23), changes in legal drug consumption (24), cardiovascular disease (25), and worsens the outcomes of many medical illnesses (26), potentially even leading to suicide (27, 28). Additionally, various physical and mental factors influence the prevalence of depressive symptoms, such as PSQ (29), bodily pain (30), and poor cognitive and physical functioning (31). Scholars have noted that there is a remarkable association between alterations in sleep patterns and depression (32). Furthermore, in the internet age, studies show that individuals who experience depressive symptoms often suffer from social media addictions, such as Facebook (33, 34), mobile phone (35), and internet addictions (8). For instance, Ivanova found that MPA was positively related to both depression and loneliness in Ukrainian students (36).

In China, the school environment and parental practices contribute to the extraordinarily high expectations of students' academic performance (37). Chinese students experience high levels of academic stress throughout their academic careers, including numerous, intense examinations—such as end-of-term tests and the standardized senior high school and university entrance examinations—and a heavy homework burden (37). Scholars have demonstrated that Chinese students experience sleep deprivation owing to this culture of academic achievement. A study of 9,392 Chinese students in primary education through university levels showed that 35.6% of participants slept <7 h a day (38). In addition to the threat of academic stress and sleep deprivation, MPA is a risk factor affecting Chinese students' physical and mental health. Mobile phones have become an integral part of students' quotidian lives—Meng's survey from December 2016 to January 2017 found that 100% of the college students had mobile phones (39)—and the prevalence of problematic mobile phone use has been found to be 28.2% among Chinese college students (40). Our study explored the correlations between perceived academic stress (PAS), MPA, sleep quality, and depression among Chinese students in middle school through college levels. Based on previous literature, our study proposed research hypotheses, and tested hypothesis by using survey data on Chinese students. To our knowledge, this was the first study to investigate relations between these variables among Chinese students by using the serial mediation model.

## Literature Review and Research Hypotheses

### Academic Stress

Academic concerns are the most important sources of chronic and sporadic stress for young people in both Western and Asian countries (41). Academic stress is defined as a student's psychological state resulting from continuous social and self-imposed pressure in a school environment that depletes the student's psychological reserves (42, 43). Students experience academic stress throughout their secondary school (41), high school (44), and university (45, 46), educational careers. Studies have shown that academic stress has been positively associated with depression (41), PSQ (24, 47), and MPA (48) among students. Jayanthi observed that, compared to adolescents who do not experience academic stress, adolescents who experienced academic stress were 2.4 times more likely to have depressive symptoms (41). Other studies have found that there is a relationship between high academic stress and PSQ (47, 49). However, scholars have not adequately addressed the adverse consequences (e.g., depression, PSQ, and MPA) of Chinese students' academic stress. Hence, we propose the following hypotheses:

*H1*: PAS is positively associated with depression.

*H2*: PAS is positively associated with MPA.

*H3*: PAS is positively associated with PSQ.

### MPA

MPA is one of the most common behavioral (i.e., non-drug) addictions (48), and is accompanied by negative effects, such as PSQ (50), depression (35), and impaired academic performance (51). The positive relationship between MPA and PSQ has been proved in previous studies, including a longitudinal study conducted among Korean adolescents (52) and a one-year prospective study among Chinese college students (50). Zhang found that among Chinese university students, there is a significant positive relationship between smartphone addiction and bedtime procrastination, which is one of the indicators of PSQ (53). Hence, we propose the following hypothesis:

*H4*: MPA is positively associated with PSQ.

Similarly, the positive relationship between MPA and depression has been proved in previous studies, including a cross-sectional study conducted among Saudi university students (35), a cross-sectional study among Ukrainian college students (36), and a systematic review of relations between problematic smartphone use, anxiety and depression psychopathology (54). Furthermore, another study based on three cohorts of Korean children and adolescents confirmed the bidirectional relationship between MPA and depression (55). Hence, we propose the following hypothesis:

*H5*: MPA is positively associated with depression.

Researchers have documented that stress is associated with MPA, and that MPA is associated with depression. For example, according to Wan et al., smartphone addictions are significantly positively associated with both depression and stress among Malaysian public university students (56). However, it is unclear if MPA mediates the relationship between PAS and depression. Hence, we propose the following hypothesis:

*H6*: MPA mediates the relationship between PAS and depression.

### Sleep Quality

Sleep disturbance has complex associations with depression (major depressive disorder) (31), and is a common physical symptom of depression. Numerous studies have confirmed the remarkable association between PSQ and depression (29, 57, 58). For example, Okun et al. found that PSQ is positively related to depression symptoms in postpartum women (29). Hence, we propose the following hypothesis:

*H7*: PSQ is positively associated with depression.

Scholars have also demonstrated that there are relationships between stress, PSQ, and depression. A prospective birth cohort study showed that PSQ is associated with stress and depression symptoms among Chinese pregnant women (58). Zhang et al. found that perceived stress is associated with sleep quality and depressive symptoms among Chinese nursing students (59). However, it has not been documented if sleep quality mediates the relationship between PAS and depression among Chinese students. Hence, we propose the following hypothesis:

*H8:* Sleep quality mediates the relationship between PAS and depression.

### Mobile Phone Addiction and Sleep Quality and the Relationship Between Perceived Academic Stress and Depression

Scholars have posited that there are significant associations between MPA, depression levels, and sleep quality. Demirci found that there were positive correlations between MPA, depression levels, and sleep quality (60). The results of Kaya's multivariate regression analysis showed a relationship between smartphone usage, PSQ, and depression in university students (57). A recent meta-analysis also found that there are positive correlations between MPA, depression, and sleep quality (61). Another literature review and case study found that depressive symptoms are associated with screen time-induced poor sleep, digital device night use, and mobile phone dependency (62). Although these studies explored the correlations between MPA, sleep quality, and depression among students, several scholars have added academic stress into the relationship—for example, a review found that sleep disturbance, anxiety, stress, and depression have been associated with problematic mobile phone use (63). There still exist gaps in the literature on how PAS influences depression. First, few scholars have focused on PAS, MPA, sleep quality, and depression among Chinese students. Second, the underlying mediating mechanisms that account for this association have been disregarded partly. Based on H6 and H8, it remains unclear if MPA and sleep quality serially mediate the relationship between PAS and depression. Therefore, we propose the following hypothesis:

*H9:* MPA and sleep quality serially mediate the relationship between PAS and depression.

### Study Objectives

In this study, our primary aim was to investigate the prevalence of depression and sleep disturbance among Chinese students. Our secondary aim was to test if there were relationships between PAS, MPA, sleep quality, and depression. First, we tested if there was a relationship between PAS and depression among Chinese students (H1: PAS is positively associated with depression). Second, we tested if MPA was a mediator of the relationship between PAS and depression (H2: PAS is positively associated with MPA, H5: MPA is positively associated with depression, and H6: MPA mediates the relationship between PAS and depression). Third, we tested if sleep quality was a mediator of the relationship between PAS and MPA (H3: PAS is positively associated with PSQ, H7: PSQ is positively associated with depression, and H8: Sleep quality mediates the relationship between PAS and depression). Finally, we also tested if MPA and sleep quality together played a serial mediating role in the influence of PAS on depression (H4: MPA is positively associated with PSQ and H9: MPA and sleep quality serially mediate the relationship between PAS and depression).

## Methods

### Survey

Data were collected from a cross-sectional questionnaire survey that was conducted from September to December 2018 in Heilongjiang Province, China, by the Heilongjiang Center for Disease Control and Prevention. A multistage cluster sampling method was used. In the first stage, three cities of Heilongjiang province were randomly selected by economic characteristics. In the second stage, one urban district and one rural township were chosen at random. In the third stage, two middle schools were randomly selected in each urban district and rural township; Since nine-year compulsory education was implemented in China, high school education is not included in the nine-year compulsory education, high schools are more in urban districts than in rural townships, two high schools and one high school were randomly selected in urban district and rural township, respectively; Since vocational high schools and universities are scarce in rural townships, one vocational high school and one college were randomly selected from the urban district. In the fourth stage, two classes were randomly selected from each grade of middle school, high school, vocational high school, and from grades 1, 2, and 3 in college. Since senior students may have been looking for a job or working as an intern, some of them were not on campus, they were not been investigated. Finally, four middle schools, three high schools, one vocational high school, and one college were randomly selected within each city (Harbin, Jiamusi, and Jixi) of Heilongjiang Province. Data were collected through a self-administered questionnaire distributed in class. Students completed the survey within 1 h, while a well-trained member of the research group supervised. All the students were informed of the purpose of the study and assured that their identities would remain confidential. Students and their parents provided written informed assent to participate in the study.

### Participants

Finally, we recruited 6,480 students in our investigation; 6,430 (99.23%) valid questionnaires were analyzed after excluding those with incomplete information. Participants were included in the sample if they had one constant internet-accessible mobile phone, which is similar with previous studies (64–68). A total of 5,109 (79.46%) participants reported having one constant internet-accessible mobile phone at the time of the survey. The final sample comprised 1,904 middle school students from grades 1, 2, and 3, respectively; 1,859 high school students from grades 1, 2, and 3, respectively; 660 vocational high school students from grades 1, 2, and 3, respectively; and 686 college students from grades 1, 2 and 3, respectively. Of these participants, there were 2,422 (47.41%) boys and 2,687 (52.59%) girls; on average, the mean age of participants was 15.53 years, with a standard deviation of 2.22, ranging from 11 to 25 years. Approval was obtained from the Medical Research Ethics Committee of Harbin Medical University and the principals of the participating schools.

### Measures

#### Perceived Academic Stress

Consistent with previous studies (69–71), PAS was measured using one self-report item “How much academic stress did you feel in the study during the past month?” using a 5-point Likert scale where 1 = “No,” 2 = “relatively low,” 3 = “average/general,” 4 =“relatively high” and 5 = “extremely heavy,” with a higher score indicating more PAS.

#### Depression

Center for Epidemiologic Studies-Depression Scale. The 20-item CES-D developed by Radloff (72) is a self-report measure that has been widely used to assess depressive symptoms in different populations (73). The reliability and validity of the CES-D have been tested among Chinese populations (74). The CES-D, when used in Chinese adolescents and university students, has shown good reliability (75–78), as well as good validity (77, 78). There are four components of CES-D, namely somatic and retarded activity, depressed affect, positive affect, and interpersonal relationships. Among the 20 items, four (items 4, 8, 12, and 16) are reversed scores. All items are evaluated on a 4-point Likert scale in relation to their incidence during the previous week, and are scored from 0 to 3 (0 = not at all, 1 = a little, 2 = some, 3 = a lot); total possible scores thus range from 0 to 60, with higher scores indicating greater number of symptoms (79).

For the original CES-D scale, a total score of 16 or greater is considered as indicative of subthreshold depression (72). Many studies have evaluated the diagnostic accuracy of the CES-D to detect depression among the general population and proposed a variety of cut-off scores, such as a cut-off score of 21 for Chinese patients with type 2 diabetes (80), and a cut-off score of 22 for the older Chinese population (81). However, the cut-off score of 16 has been widely used for Chinese adolescents and university students (7, 76, 82–84). Therefore, the same cut-off score has been used in our study too. Students with CES-D scores between 16 and 21 were defined as “mildly depressed,” between 21 and 24 as “moderately depressed,” and ≥ 25 as “severely depressed” (83). The CFA on the four-factor model showed a good model fit, with χ^2^ = 16.54, df = 1, *P* < 0.000, RMSEA = 0.06, SRMR = 0.01, CFI = 0.99, TLI = 0.98. Additionally, the Cronbach's alpha coefficient was 0.84 for the total scale, all four dimensions had acceptable reliability with Cronbach's alpha coefficient of 0.70, 0.83, 0.78, and 0.62.

#### Mobile Phone Use Situation and Mobile Phone Addiction

Mobile phone use situation was assessed by three items. First, “How many hours do you use your mobile phone every day?” to which participants answered with one of four options: “less than a half hour,” “a half hour to one hour,” “one to two hours,” or “more than two hours.” Second, “How long have you had a mobile phone?” to which participants answered “ <1 year,” “1–2 years,” “2–3 years,” or “more than 3 years.” Third, “How much do you spend on mobile phone charges every month?” to which participants answered “less 30 yuan,” “30–50 yuan,” “50–100 yuan,” or “more than 100 yuan.”

The Mobile Phone Addiction Index (MPAI) was used in our study (85). Participants rated the 17 items on a 5-point Likert scale ranging from 1 (not at all) to 5 (always). Higher scores indicated greater addiction to mobile phones (86). There are four components of MPAI, namely inability to control craving, feeling of anxiety and being lost, withdrawal or escape, and productivity loss. The Confirmatory Factor Analysis (CFA) on the four-factor model showed a good model fit, with χ^2^ = 6.44, df = 1, *p* < 0.05, RMSEA = 0.03, SRMR = 0.004, CFI = 0.99, TLI = 0.99. Additionally, the Cronbach's alpha coefficient was 0.90 for the total scale. All four dimensions had satisfactory reliability with Cronbach's alpha coefficient of 0.76, 0.81, 0.85, and 0.75.

### Sleep Quality

The Pittsburgh Sleep Quality Index (PSQI) was used in our study (87). PSQI scale contains 19 items covering seven components: subjective sleep quality, sleep latency, sleep duration, habitual sleep efficiency, sleep disturbances, use of sleep medication, and daytime dysfunction. Each component was scored from 0 (no difficulty) to 3 (severe difficulty). The total score was calculated from the seven component scores, ranging from 0 to 21. A score of more than 5 implied poor sleep (87). The CFA on the seven-factor model showed a good model fit, with χ^2^ = 79.49, df = 11, *P* < 0.000, RMSEA = 0.04, SRMR = 0.02, CFI = 0.99, TLI = 0.98. Additionally, Cronbach's alpha coefficient was 0.624 for the PSQI scale in our study.

### Data Analyses

SPSS version 19.0. and Mplus 7.0 were used to analyse data in our study. Descriptive analyses were first conducted of participants' characteristics, participants' mobile phone use and the prevalence of sleep disturbance, MPA, and depression. We tested the reliability and validity of the MPAI scale, PSQI scale and CES-D scale by examining their Cronbach's alpha coefficient and performing a CFA. Spearman's correlation analysis was performed to examine the general relationships among the four variables—PAS, MPA, sleep quality, and depression. A structural equation model (SEM) was built to examine hypotheses. We tested the mediating role of MPA and sleep quality; the constructed serial mediation model included three latent variables (MPA, sleep quality and depression) and one manifest variable (PAS), PAS was the independent variable, depression was the dependent variable, and MPA and sleep quality were the mediating variables (88). The bootstrapping analyses used 5,000 samples at the 95% confidence interval (CI) to indicate significance.

To determine whether the model fits the data well, multiple indices were tested, including (1) the model χ^2^ and its *p* value, in which non-significance is desirable for good fit. With increasing sample size and a fixed degree of freedom, the χ^2^ value increases. It is difficult to get a nonsignificant chi-square (indicative of good fit) when sample sizes are over 200 (89). This can lead to a problem where plausible models might be rejected. Because this statistic is sensitive to the sample size, inspection of the other fit indices is recommended (90). (2) The root mean square error of approximation (RMSEA) in which values ranging from 0.05 to 0.08 represent adequate fit, and values <0.05 indicate good fit. (3) The standardized root mean square residual (SRMR) in which values are ≤0.08 indicate good fit. (4) The comparative fit index (CFI), in which values range from 0.90 to 0.95 indicate an adequate fit and values ≥0.95 indicate a good fit, and (5) the Tacker-Lewis index (TLI) in which values >0.90 indicate a good fit.

## Results

### Descriptive Statistics

The mean scores of PAS were 2.61 ± 1.03, 2.68 ± 1.06, 2.13 ± 0.98, and 2.29 ±0.96 for middle school students, high school students, vocational high school students, and college school students, respectively.

Among the participants, 45.55% used their mobile phone more than 2 h daily; 39.5% of the participants had a mobile phone for more than 3 years; 53.24% of the participants spent more than or equal to 30 yuan on mobile phone charges every month (Table 1). The mean MPAI score of all the participants was 30.62 ± 11.92.

**Table 1:** Participants' mobile phone use.

	**N**	**%**
**Daily usage time of mobile phone**		
< a half hour	195	3.82
A half hour to 1 h	1,108	21.69
1–2 h	1,479	28.95
More than 2 h	2,327	45.55
**Duration of owing a mobile phone**		
<1 year	842	16.48
1–2 years	1,320	25.84
2–3 years	929	18.18
More than 3 years	2,018	39.5
**Mobile phone charges every month**		
<30 yuan	2,389	46.76
30–50 yuan	1,942	38.01
50–100 yuan	574	11.24
More than 100 yuan	204	3.99

The prevalence of depressive symptoms was 28.69% (*n* = 1,466) with a mean CES-D score of 12.52 ± 8.86. Prevalence of depression at a mild level (CES-D ≥ 16 and CES-D < 21), moderate level (CES-D ≥ 21 and CES-D < 25), and severe level (CES-D ≥ 25) was 12.62, 6.95, and 9.12%, respectively. The prevalence of depressive symptoms among high school students (33.14%) was the highest, while the prevalence of depressive symptoms among college students (21.43%) was the lowest (Table 2).

**Table 2:** Depression classifications of participants.

	**Classifications**	**N**	**%**
Depression	No (CES-D < 16)	3,643	71.31
	Mild (CES-D ≥ 16 and CES-D < 21)	645	12.62
	Moderate (CES-D ≥ 21 and CES-D < 25)	355	6.95
	Severe (CES-D ≥ 25)	466	9.12
Learning stage	Middle school	508	26.68
	High school	616	33.14
	Vocational high school	195	29.55
	College	147	21.43
All participants		1,466	28.69

The prevalence of sleep disturbance was 27.95% (*n* = 1,428) with a mean global PSQI score of 4.29 ± 2.59. The prevalence of sleep disturbance among high school students (36.47%) was the highest, while the prevalence of sleep disturbance among middle school students (20.75%) was the lowest. The average sleep time and sleep latency were 7.40 ± 1.28 h and 15.81 ± 12.48 min, respectively. Among the participants, 14.50% reported that they had bad or very bad sleep quality; 36.29% reported that their sleep latency was more than 15 min; 50.89% reported that they slept ≤7 h a day; 12.62% reported that their sleep efficiency was ≤85%; 67.59% reported that they experienced sleep disturbances; 2.90% of them reported that they used sleep medication; and 78.80% reported that they had daytime dysfunction (Table 3).

**Table 3:** Prevalence of sleep disturbance for participants at different education levels.

**Learning stage**	**N**	**%**
Middle school	395	20.75
High school	678	36.47
Vocational high school	177	26.82
College	178	25.95
All participants	1,428	27.95

Means, Standard Deviation (SD) and correlations of the main variables in the mediation model are shown in Table 4. The results, indicating that the variables were significantly and positively correlated, provide initial support for the hypotheses of this study, and act as a foundation of the serial mediation model.

**Table 4:** Means, SD, Pearson's correlation coefficient of variables.

	**M**	**SD**	**1**	**2**	**3**	**4**
1. PAS	2.53	1.04	1			
2. MPAI global score	30.62	11.92	0.164^*^	1		
3. PSQI global score	4.29	2.59	0.253^*^	0.401^*^	1	
4. CES-D global score	12.52	8.85	0.236^*^	0.327^*^	0.410^*^	1

* *p ≤ 0.01*.

### Test for Serial Mediation Model

SEM was used to provide the fit indexes of the serial mediation model. A model was constructed with MPA (M1) as a mediator and sleep quality (M2) as another mediator. In this model, PAS was set as the predictor (X) and depression as the outcome (Y). Results of the serial mediation model indicated that the constructed model exhibited a satisfactory fit with the data: χ^2^ = 1,196.50, df = 95, *P* < 0.000, SRMR = 0.04, RMSEA = 0.05, CFI = 0.95, and TLI = 0.94.

First, PAS was positively associated with depression (B = 0.10, SE = 0.02, 95% CI = 0.06–0.13). Higher levels of PAS were related to higher levels of depression, and thus H1 was supported. Second, PAS positively predicted MPA (B = 0.18, SE = 0.02, 95% CI = 0.15–0.21). Higher levels of PAS were related to higher levels of MPA, and thus H2 was supported. Third, PAS was positively associated with PSQ (B = 0.23, SE = 0.02, 95% CI = 0.19–0.26). Higher levels of PAS were related to poorer sleep quality, and thus H3 was supported. Fourth, MPA was positively associated with PSQ (B = 0.51, SE = 0.02, 95% CI = 0.47–0.54). Higher levels of MPA were related to poorer sleep quality, and thus H4 was supported. Fifth, MPA was positively associated with depression (B = 0.17, SE = 0.02, 95% CI = 0.13–0.22). Higher levels of MPA were related to higher levels of depression, and thus H5 was supported. Last, PSQ was positively associated with depression (B = 0.44, SE = 0.03, 95% CI = 0.39–0.49). PSQ was related to higher levels of depression, and thus H7 was supported.

### Total, Direct, and Indirect Effects

Table 5 shows all possible indirect effects of the mediation model. First, the indirect effect of PAS on depression through MPA was significant (B = 0.08, 95% boot CI = 0.06–0.11), and thus H6 was supported. Second, the indirect effect of PAS on depression through sleep quality was significant (B = 0.27, 95% boot CI = 0.22–0.33), and thus H8 was supported. Third, the indirect effect of PAS on depression through MPA and sleep quality was also significant (B = 0.11, 95% boot CI = 0.08–0.14), and thus H9 was also supported. The total indirect effect was B = 0.46, 95% boot CI = 0.40–0.53, and the mediating effect of MPA and sleep quality were significant (*P* < 0.001), accounting for 64.01% (total indirect effect/total effect) of the total effect. The indirect effect related to sleep quality accounted for 82.61% of the total indirect effect, that is, (indirect effect 2 + indirect effect 3)/total indirect effect (Table 5).

**Table 5:** Total, direct, and indirect effects.

	**Depression outcomes as criterion**				
	**B**	**SE**	**t**	**LLCI**	**ULCI**
Total effect of PAS on depression	0.71	0.05	15.11	0.63	0.81
Total direct effect of PAS on depression	0.26	0.05	5.75	0.17	0.35
Total indirect effect of PAS on depression	0.46	0.03	13.47	0.40	0.53
Indirect effect 1: PAS→ MPA→ depression	0.08	0.01	5.92	0.06	0.11
Indirect effect 2: PAS→ sleep quality→ depression	0.27	0.03	9.50	0.22	0.33
Indirect effect 3: PAS→ MPA→ sleep quality→ depression	0.11	0.01	8.16	0.08	0.14

*Number of bootstrap samples for bias-corrected bootstrap CIs: 5,000. Level of confidence for all CIs: 95*.

*CI, confidence interval; LLCI, low limit confidence interval; ULCI, upper limit confidence interval*.

## Discussion

Although academic stress is a well-known risk factor for depression in students, little is known about the possible psychological mechanisms underlying this association, or how MPA and PSQ—which also are risk factors of depression—operate to have an impact on it. The main aim of our study was to test if there is a relationship between PAS and depression and if MPA and sleep quality together play a serial mediating role in the influence of PAS on depression among Chinese students. To the best of our knowledge, this was the first study to investigate the relationship between the variables using SEM. As expected, the serial mediation model showed that PAS was a significant predictor of depression. MPA and sleep quality played a mediating role between PAS and depression. Furthermore, MPA and sleep quality together played a serial mediating role in the influence of PAS on depression. In our study, the indirect effect (i.e., the mediating effect of MPA and sleep quality) was significant and accounted for 64.01% of the total effect. Thus, apart from the direct effect of PAS on depression, the indirect effect of PAS on depression should be emphasized. Our findings provide significant insights into the risk factors for depressive symptoms in students.

### Depression Among Students

According to studies that have focused on depression among Chinese students, the prevalence of depression varies from 22.0 to 68.5% (5, 91–95). In our study, the prevalence of depressive symptoms was 28.69%. The differences across these studies may have resulted from temporal or regional disparities or variations in depression definitions and assessment methods. Depressive symptoms are related to many negative consequences, such as increased suicide risk among students (96) and increased college withdrawal rates (97). Controlling depressive symptoms among students can both protect human capital value from the societal perspective and maintain students' physical and mental health from the individual perspective. In our study, the most stressed, depressed, and sleep-deprived students were high school students. Thus, Chinese high school students' physical and mental health requires attention. In China, high school students are admitted to colleges and universities based on *gaokao*, the standardized National College Entrance Examination (98). These admission decisions are extremely important, as they impact high school students' future educational opportunities, career paths, and life experiences. Our research results prove that Chinese students experience the most stressful and competitive academic environment of their academic careers when they are in high school.

### Mediating Role of Mobile Phone Addiction

Chinese students spend considerable time on mobile phones−45.55% of the participants spent more than 2 h daily on their mobile devices. 39.5% of participants had had a mobile phone for more than 3 years, while the mean age of participants was 15.53 years. Using the mediation model, we illustrated the mediating role of MPA in line with our hypothesis. As H6 predicted, MPA played a role in the path from PAS to depression. MPA could partially explain the association between PAS and depression among Chinese students—hence, MPA was not only an outcome of PAS, but also a catalyst of depression. First, we found that high levels of PAS were associated with high levels of MPA. This finding is consistent with previous research results (48) and suggests that PAS may be a significant trigger for students' negative behaviors—such as MPA. Scholars have posited that young people's digital distraction activities—including playing computer games and online surfing—may be interpreted as a way to avoid problems, reality, and stress (99, 100). High levels of PAS were associated with high levels of MPA, which may be due to students' use of mobile phones to escape from academic stress. Second, we found that high levels of MPA were associated with high levels of depression, which is in line with existing research results (35, 36, 101). Students who experience MPA may neglect real-world social engagement (102) resulting in academic underperformance (103), clinical health symptoms (68), which are related to negative emotions—such as depression. Our findings add to the existing research that suggests that when students are facing academic stress, they may be addicted to their mobile phones to escape from academic stress, and thus the negative consequences of MPA may lead to depression in students.

### Mediating Role of Sleep Quality

As H8 predicted, sleep quality is not only an outcome of academic pressure—it is also a catalyst of depression. Moreover, the indirect effect related to sleep quality accounted for 82.61% of the total indirect effect. Thus, compared to MPA, sleep quality played a more important role in the path from PAS to depression. We found that higher levels of PAS were associated with poorer sleep quality. This finding is consistent with previous research findings (24, 47). For example, Waqas et al. demonstrated that perceived stress is a significant predictor of PSQ (47). In China, students exist in a prolonged competitive learning environment and experience unrelenting academic stress. To achieve better academic performance and meet the extraordinarily high expectations of parents and educators, Chinese students have heavy homework burdens and learning burdens, resulting in sleep deprivation. Furthermore, academic stress decreases sleep quality. According to Almojali et al., students who are not suffering from academic stress are less likely to experience PSQ (104). Previous studies have proved that sleep deficiency and sleep health problems are common among Chinese students (105). Our research results may explain why higher levels of PAS were related to poorer sleep quality.

We also found that high levels of PSQ were associated with high levels of depression, which is consistent with prior research findings (31, 50, 57). Scholars have proved that PSQ is related to multiple negative consequences that may lead to depression—including daytime dysfunction, poor academic performance, and fatigue (106, 107). Our findings add to the existing research that suggests that sleep quality is a mediator between PAS and depression among students, which means that higher levels of PAS were related to poorer sleep quality—such as sleep deficiency and daytime dysfunction—which was related to higher levels of depression.

### Serial Mediating Effect of Mobile Phone Addiction and Sleep Quality

As per H9, MPA and sleep quality together play a serial mediating role in the influence of PAS on depression. The results of our study showed that higher levels of PAS were related to higher levels of MPA, which was associated with poorer sleep quality, which was associated with higher levels of depression. Numerous studies have documented that there is a positive relationship between MPA and PSQ (50, 52). For example, Kang et al. found that there were bidirectional longitudinal relationships between MPA and PSQ (50). Scholars have posited that the more screen time young people use, the less sleep time they have (108). Moreover, young people often use their mobile phone in the bedroom—bedtime mobile phone use is related to higher insomnia scores and increased fatigue (109), and both insomnia and fatigue are related to depression (110, 111). This may explain why MPA and PSQ together play a serial mediating role in the influence of PAS on depression. Our findings suggests that Chinese students are likely to distract themselves from PAS by using their mobile phones, and thus shortening their sleep duration, decreasing their sleep quality, leading to PSQ, and resulting in depressive symptoms.

### Measures to Reduce Depressive Symptoms Among Chinese Students

To reduce depressive symptoms among students, their PAS should be managed. Given the multiple, negative consequences (MPA, PSQ, and depression) of PAS, stakeholders—family members, educators (including teachers, school administrators, and school health professionals), and policy makers—should take preventative measures to help students manage and relieve academic stress, such as provide counseling services (112), foster their psychological resilience (113), and increase social support (19) to improve their overall well-being. Second, students' sleep quality should be ensured to reduce depressive symptoms. Stakeholders should actively promote counseling and intervention for students experiencing sleep disturbances. Third, given that higher levels of MPA are associated with poorer sleep quality and higher levels of depression, stakeholders should develop mitigating strategies to manage mobile phone use to ensure students' sleep quality and to relieve their depressive symptoms. Rational and normative mobile phone use should be advocated and classroom management strategies enforced to ensure that students use their mobile phones at restricted times and places for positive purposes, such as online learning. Fourth, regular psychological assessment of depression, MPA, and PSQ will help stakeholders detect and manage students' health problems. Last, parents and family members, educators, and policy makers should encourage students to exercise more to alleviate MPA (114), improve sleep quality (115), and reduce depression (116).

### Limitations

This study has several limitations. First, although we conducted our research based on previous studies, due to the cross-sectional design of our study, we could not confirm causal relationships among the study variables. Second, the study period was September to December 2018, which was more than 2 years ago. However, we believe that the results of our study are valuable for understanding the mechanisms of how PAS influences students' depression through MPA and sleep quality, and our study can provide a basis for future research. Third, the study measured the participants' perceived academic stress using a single item, which may not have captured various other relevant stressors, such as parental learning expectations. Future studies should use a multiple-item scale to assess the participants' perceived academic stress. Forth, this study was limited to middle school, high school, vocational high school, and college students. Future research on Chinese students at all education levels from primary school to postgraduate levels is necessary. Fifth, perceived academic stress can increase during stressful conditions (117), such as during exams or major change in life (e.g., from high-school student to freshman). While our study was conducted in 27 schools in three cities, it is impossible that we conducted the survey when the participants had no examinations or changes. Future studies can control for stressful academic conditions in the analyses to enhance their accuracy. Last, gender, age, and other factors are important influencing factors of PAS, MPA, sleep quality, and depression. Since the main aim of this study was to test if there was a relationship between PAS and depression and if MPA and sleep quality together play a serial mediating role in the influence of PAS on depression, the aforementioned factors were not considered in this study. Future studies should consider these factors and test the relationships between PAS, MPA, sleep quality, depression, and other health indicators.

## Conclusions

Our study's results showed that Chinese students face the risk of depression and sleep disturbance, and the most stressed, depressed, and sleep-disturbed students are those in high school. Second, the results of the serial mediation model indicated that PAS predicted depression, and MPA and sleep quality played a mediating role between PAS and depression. Furthermore, MPA and sleep quality together play a serial mediating role in the influence of PAS on depression. Our study extends the understanding of how PAS is associated with depression among Chinese students. Considering the harmful effects of depression, stakeholders—including parents and family members, educators, and policy makers—should take preventative measures to alleviate Chinese students' depression and depressive symptoms.

## Data Availability Statement

The raw data supporting the conclusions of this article will be made available by the authors, without undue reservation. Requests to access these datasets should be directed to wuqunhong@163.com.

## Author Contributions

XZ, FG, ZK, and QW: conceptualization. XZ, HZ, JW, and HL: formal analysis. FG, JZ, JL, JY, HZ, and BL: investigation. XZ, FG, ZK, and BL: data curation. XZ, FG, and ZK: writing—original draft preparation. QW and BL: writing—review and editing. All authors have read and agreed to the published version of the manuscript.

## Funding

This research was funded by QW of The National Key Social Science Fund of China (Grant No.19AZD013).

## Conflict of Interest

The authors declare that the research was conducted in the absence of any commercial or financial relationships that could be construed as a potential conflict of interest.

## Publisher's Note

All claims expressed in this article are solely those of the authors and do not necessarily represent those of their affiliated organizations, or those of the publisher, the editors and the reviewers. Any product that may be evaluated in this article, or claim that may be made by its manufacturer, is not guaranteed or endorsed by the publisher.

## Abbreviations

PAS: Perceived Academic Stress
MPA: Mobile Phone Addiction
MPAI: Mobile Phone Addiction Index
CES-D: Center for Epidemiologic Studies-Depression
PSQ: Poor Sleep Quality
PSQI: Pittsburgh Sleep Quality Index
SEM: Structural Equation Model.
